# Influence of endurance exercise training on antioxidant enzymes, tight junction proteins, and inflammatory markers in the rat ileum

**DOI:** 10.1186/s13104-015-1500-6

**Published:** 2015-09-30

**Authors:** A. Maleah Holland, Hayden W. Hyatt, Ashley J. Smuder, Kurt J. Sollanek, Aaron B. Morton, Michael D. Roberts, Andreas N. Kavazis

**Affiliations:** School of Kinesiology, Auburn University, 301 Wire Road, Auburn, AL 36849 USA; Department of Applied Physiology and Kinesiology, University of Florida, Gainesville, FL USA

**Keywords:** Exercise, Antioxidants, Oxidative stress

## Abstract

**Background:**

This study investigated the effects of endurance exercise training on ileum antioxidant status, as well as tight junction, inflammatory, and nutrient transporter gene expression.

**Methods:**

Sprague–Dawley rats (4 month old) were assigned to sedentary (SED) or endurance exercise-training (EXE) groups (n = 8/group). EXE animals were trained on the treadmill for 10 days at a speed of 30 m/min at 0° incline for 60 min/day. SED and EXE animals were sacrificed (24 h after the final training bout) and the ileum was stored for analyses.

**Results:**

The ileum of EXE had higher (p < 0.05) antioxidant protein levels of manganese superoxide dismutase and catalase compared to SED with no change (p > 0.05) in the lipid peroxidation biomarker 4-hydroxynonenal. Ileum mRNA expression of the tight junction gene zonulin increased (p < 0.05) and claudin 1 decreased (p < 0.05) in EXE compared to SED, but occludin and zonula occluden 1 were not different (p > 0.05) between SED and EXE. The ileum mRNA expressions of seven nutrient transporters (SLC5A8, SLC7A6, SLC6A19, SLC7A7, SLC27A2, SLC16A10, and SLC15A1) were not different between the two groups (p > 0.05). EXE had lower ileum TNFα mRNA expression (p < 0.05) compared to SED. No changes (p > 0.05) were found in the other inflammatory mRNAs including NFκB, IFNγ, IL6, CCL2, TLR4, and IL10. In addition, no changes in p-p65:p65 were detected.

**Conclusions:**

These findings suggest that 10 days of endurance exercise training up-regulates key endogenous antioxidant enzymes, decreases select inflammation markers, and alters select markers of tight junction permeability.

## Background

Regular exercise results in physiological adaptations that benefit almost every organ system of the body, improving overall health. While the effect of exercise on the gastrointestinal (GI) system is still controversial [1–3], it appears that exercise intensity plays an important role in the relationship between exercise and gut health [1, 4]. Athletes participating in long duration and intense exercise (i.e., marathon running) can experience GI symptoms such as diarrhea and intestinal bleeding [1]. However, the exact mechanisms of exercise-induced GI distress are unknown. In this regard, intestinal ischemia–reperfusion occurs during exercise and recovery [5]. It is postulated that splanchnic vasoconstriction from strenuous exercise may disturb the pro-oxidant to antioxidant balance. Evidence suggests that this is due to a large generation of intracellular free radicals which overwhelms the antioxidant defense system, thus resulting in oxidative stress and possibly tissue damage [6]. Specifically, Rosa and collaborators revealed that 10 or more days of running at 85 % maximal oxygen consumption to exhaustion increased oxidative stress in the ileum and significantly damaged the ileum mucosa layer, possibly compromising intestinal barrier integrity [7]. However, it is important to note that several factors (e.g., mode, duration, and intensity of exercise) can have either additional benefits or detriments to the gut (reviewed in [1]).

Intestinal ischemia may augment intestinal barrier permeability via oxidative stress accumulation [6]. Hydrogen peroxide, a known reactive oxygen species, causes tissue breakdown and activates nuclear factor kappa-B (NFκB) which initiates transcription of several pro-inflammatory cytokines including tumor necrosis factor-α (TNFα), interleukin 6 (IL6), interferon-γ (IFNγ), and interleukin 1β (IL1β) [2]. This cascade results in the disruption of tight junction proteins resulting in enhanced intestinal permeability. An increase in intestinal permeability allows for the passage of endotoxins into circulation which may lead to local and possibly systemic inflammatory responses [8]. Therefore, the aim of the present study was to investigate: (1) ileum antioxidant capacity, (2) gene markers related to intestinal barrier integrity, (3) gene markers of ileum inflammation, and (4) nutrient transporter gene adaptations in the ileum that occur in response to a global physiological challenge (e.g., endurance exercise). We hypothesized that endurance exercise training for 10 days would increase endogenous antioxidant protein levels and modify tight junction, inflammatory, and nutrient transporter gene expression in the ileum.

## Methods

### Animals

Adult female Sprague–Dawley rats (4 months old) were assigned to a sedentary (SED) or endurance exercise-training (EXE) group (n = 8 per group). The animals were housed on a 12-h: 12-h light–dark cycle (20–22 °C) and provided food and water ad libitum throughout the experiment. The University of Florida Institutional Animal Care and Use Committee approved the use of animals in this experiment.

### Experimental design

The EXE animals were familiarized to treadmill running for five consecutive days (10, 20, 30, 40, and 50 min of exercise/day, respectively). Following 2 days of rest, EXE animals were trained on the treadmill for 10 days at a speed of 30 m/min at 0° incline, estimated work rate of 70 % maximum oxygen consumption [9], for 60 min per day. SED and EXE animals were sacrificed 24 h after the final training bout and the ileum (last part of the small intestine and vital organ for nutrient absorption) was stored and used for analyses.

### Western blot analysis

Western blot analysis determined protein abundance in the ileum tissue. Briefly, ileum tissue samples were homogenized 1:10 (wt/vol) in 5 mM Tris (pH 7.5) and 5 mM EDTA (pH 8) with a protease inhibitor cocktail (Sigma, St. Louis, MO) and centrifuged at 1500×*g* for 10 min at 4 ℃. The supernatant was collected and ileum protein content was assessed by the Bradford method. Proteins from the supernatant fraction of the ileum homogenates were separated via polyacrylamide gel electrophoresis (C.B.S. Scientific Company, San Diego, CA). After electrophoresis, the proteins were transferred to polyvinylidene difluoride membranes (Ameresco, Solon, OH) via the C.B.S. Scientific Company system for 2 h at 200 mA. Non-specific sites were blocked for 1 h at room temperature in PBS solution containing 0.05 % Tween and 5 % non-fat milk. Membranes were then incubated for 1 h with primary antibodies directed against the proteins of interest. The primary antibodies used were: superoxide dismutase 2 (SOD2; # GTX116093; GeneTex, Irvine, CA), catalase (GeneTex, # GTX110704), 4-hydroxynonenal conjugated proteins (4-HNE, # ab46545, Abcam, Cambridge, MA), p-p65 (Cell Signaling, Danvers, MA, # 3033) and p65 (Cell Signaling, # 8242). Following incubation with primary antibodies, membranes were washed extensively with PBS-Tween and then incubated with secondary antibodies. Membranes were then developed using an enhanced chemiluminescent reagent (Amersham, Pittsburgh, PA), and band densitometry was performed through the use of a gel documentation system and associated densitometry software (UVP, LLC, Upland, CA). Alpha tubulin (# 12G10, Developmental Studies Hybridoma Bank, Iowa City, IA) was used as the normalizing control for SOD2 and catalase. For 4-HNE, the whole lane was quantified and normalized to Ponceau S.

### RT PCR for ileum mRNA expression

RNA was isolated from ileum using the Ribozol method (Ameresco) according to the manufacturer’s instructions. Concentration and purity of the extracted RNA were measured spectrophotometrically at 260 and at 280 nm using the NanoDrop Lite Spectrophotometer (Thermo Fisher Scientific, Waltham, MA). Following isolation, 1 μg of RNA was reverse transcribed into cDNA using a cDNA synthesis kit (Quanta, Gaithersburg, MD) per manufacturer’s recommendations. Real-time PCR was performed by utilizing the CFX Connect instrument (Hercules, CA) and SYBR green chemistry (Quanta) with the gene-specific primers listed in Table 1. Tested genes were: occludin (OCLN), claudin 1 (CLDN1), zonula occluden-1 (ZO1), haptoglobin/zonulin (Zonulin), nuclear factor of kappa light polypeptide gene enhancer in b-cells (NF_K_B), tumor necrosis factor (TNFα), gamma interferon (IFNγ), interleukin 6 (IL6), chemokine (C–C motif) ligand 2 (CCL2), toll-like receptor 4 (TLR4), interleukin 10 (IL10), solute carrier family 15 member 1 oligopeptide transporter (SLC15A1), solute carrier family 6 member 19 neutral amino acid transporter (SLC6A19), solute carrier family 7 member 6 amino acid transporter light chain, y + L system (SLC7A6), solute carrier family 7 member 7 amino acid transporter light chain, y + L system (SLC7A7), solute carrier family 16 member 10 aromatic amino acid transporter (SLC16A10), solute carrier family 5 member 8 sodium/monocarboxylate co-transporter (SLC5A8), solute carrier family 27 member 2 fatty acid transporter (SLC27A2), and solute carrier family 6 member 4 sertonin neurotransmitter transporter (SLC6A4). Beta-glucuronidase (GUSB) expression was not different between the two groups and was used as the reference gene. Relative quantification of gene expression was performed using the 2∆∆CT method whereby ∆CT [CT(reference gene) − CT(gene of interest)].

**Table 1:** Gene-specific primers used for RT-PCR

Gene	Forward primer (5′ → 3′)	Reverse primer (5′ → 3′)
OCLN	CCCAGGTGGCAGGTAGATTA	AGGCCTGTTTTGTGAGCACT
CLDN1	TGTGCCACACAAACATCCTT	GGGCTCATTCCTGTCATCAT
ZO1	GTGCTCACCAGGGTCAAAAT	GGCTTAAAGCTGGCAGTGTC
ZONULIN	ACTGGGTCCAGGAAACAATG	TCCTCTTCCAGGGTGAATTG
NFkB1	TTGTCACTGCTGTCCCTCTG	GTGGGGACTGCGATACCTTA
TNFα	GGTCAACCTGCCCAAGTACT	CTCCAAAGTAGACCTGCCCG
IFNγ	AGCCTAAGGAAGCGGAAAAG	GGCACACTCTCTACCCCAGA
IL6	ATCTGCCCTTCAGGAACAGC	GAAGTAGGGAAGGCAGTGGC
CCL2	TGATCCCAATGAGTCGGCTG	ACCTGCTGCTGGTGATTCTC
TLR4	GTTGGATGGAAAAGCCTTGA	GGCGCAGAGTTTTGTACTCC
IL10	GAATTCCCTGGGAGAGAAGC	TTCTCACAGGGGAGAAATCG
SLC15A1	AGCAGAGATCGAGGCACAGT	TTCCCTACGCCCTTTTTCTT
SLC6A19	ACTGTGGTCGGTGCTCTTCT	TGAGATCCTGAAGGGGTACG
SLC7A6	TTGGGTATCATGGACAGCAA	AGGGAGGGAGTGTTGTTCCT
SLC7A7	CTCGGAACTTGCTTTTGAGG	CCGAAACAACACGTAGCAAA
SLC16A10	TGCAGCTGTAGGATTCGTTG	GCAGGCAAATACGACTCCAT
SLC5A8	TTGGTGCTGGACATTTTGAA	CTGTAAGCACAGGCCACAAA
SLC27A2	GCCCATGACTGAGGACATTT	TTAGGAGCCAGGCAACATTC
SLC6A4	CCTCTAAGCCAAGCCTGATG	AAGTGGTCGGAATCCACAAG
GUSB	CCAGAGCGAGTATGGAGCAG	CCTCACTGAACATGCGAGGT

### Statistical analysis

Dependent variable comparisons between groups were made by independent t tests with the significance set at p < 0.05. Data are presented as mean ± SE.

## Results

### Oxidative stress

**Fig. 1:**
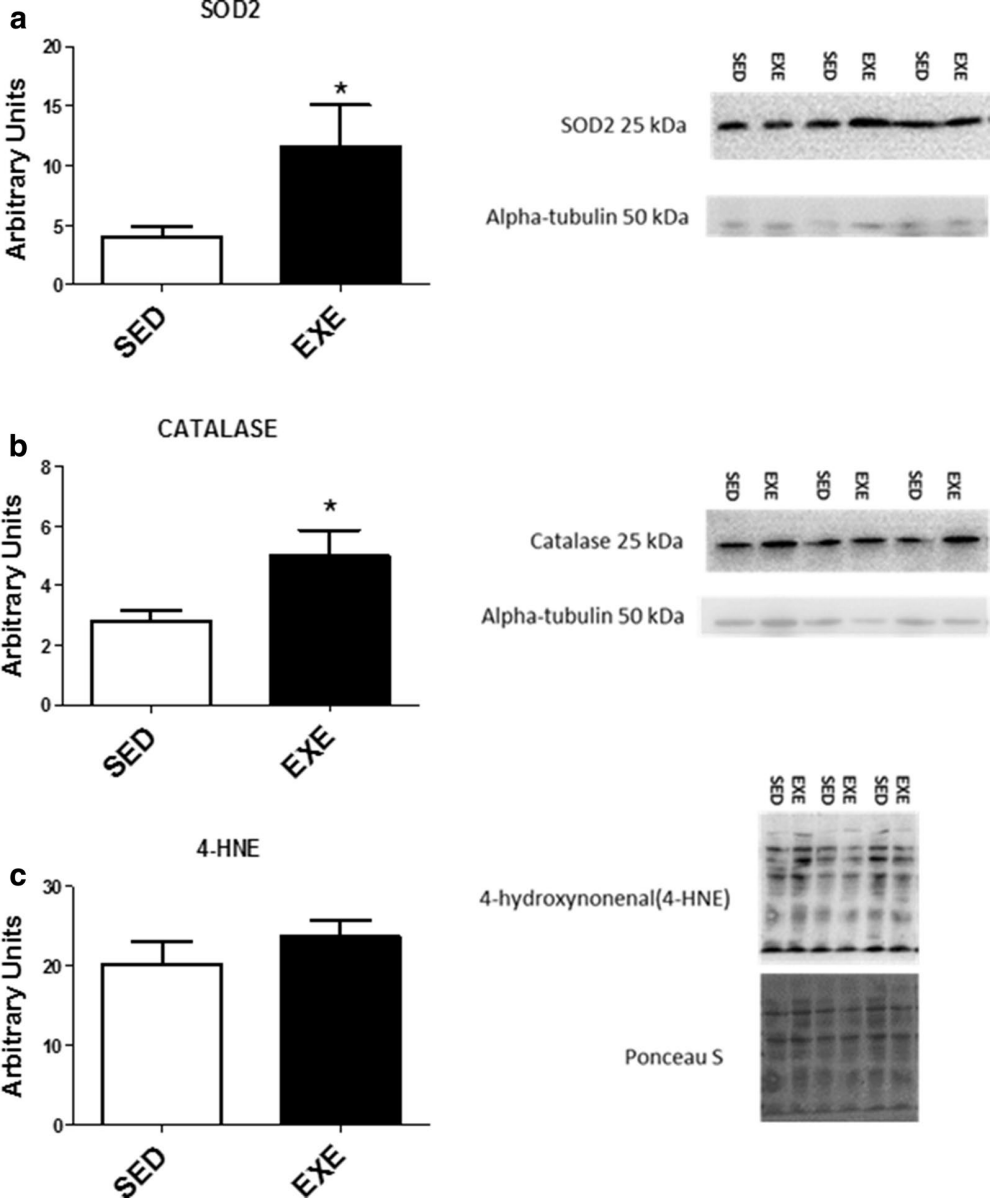
Ileum oxidative stress. **a** Manganese superoxide dismutase (SOD2), and **b** catalase protein levels in ileum tissue of sedentary and exercised animals. **c** Lipid peroxidation was compared by using 4-hydroxynonenal (4-HNE). Representative Western blots are shown to the* right* of the graphs (n = 8 per group). *p < 0.05

To assess the effects of moderate exercise training on the antioxidant capacity of the ileum, protein levels of the antioxidant enzymes, SOD2 and catalase, were evaluated. As illustrated in Fig. 1a, b, 10 days of exercise training increased SOD2 and catalase levels (p < 0.05). Additionally, a biomarker of lipid peroxidation, 4-HNE, was measured as a marker of oxidative damage. Figure 1c illustrates the levels of 4-HNE in the ileum were not different between SED and EXE groups (p > 0.05).

### Gene expression and protein levels of p-p65:p65

**Fig. 2:**
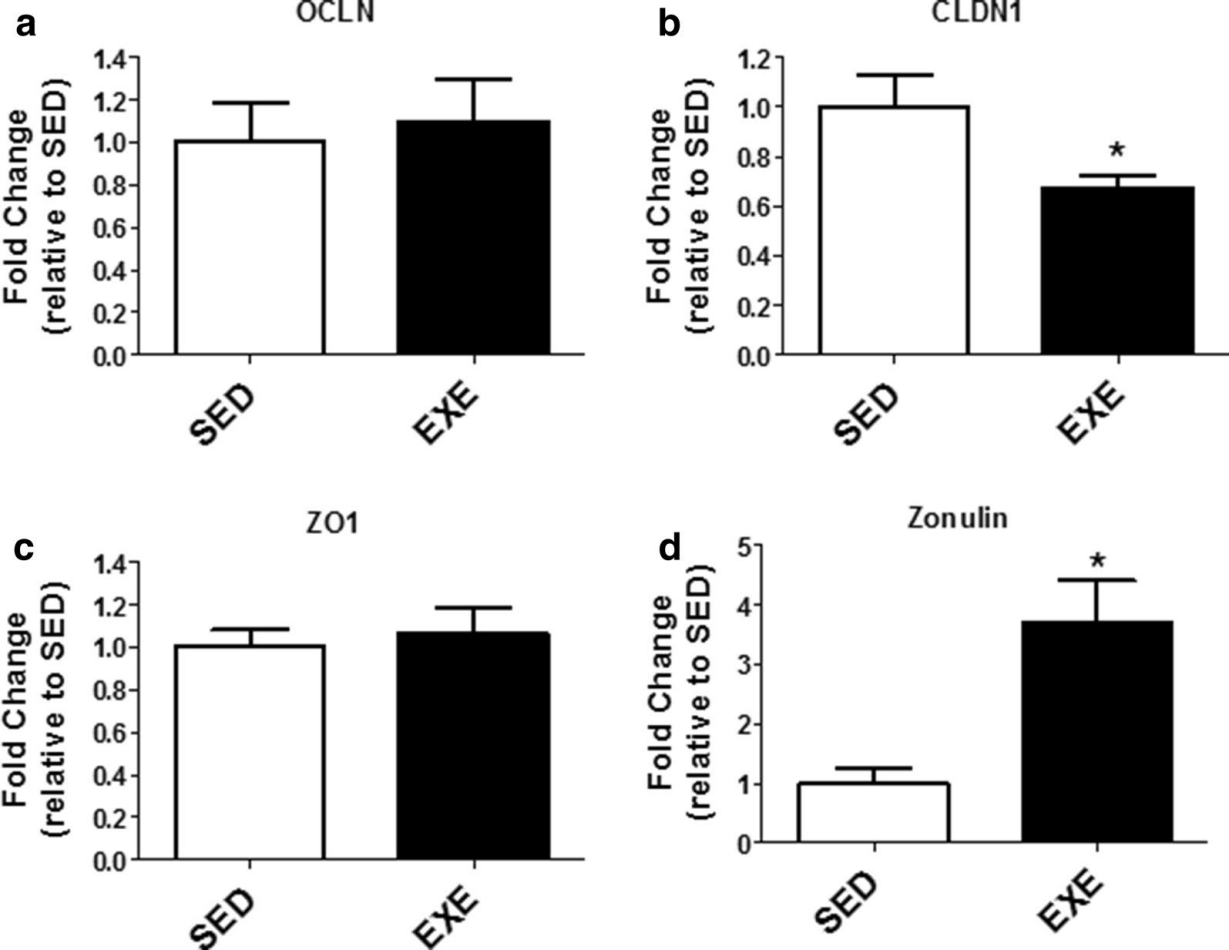
Expression of tight junction genes in the ileum. **a** Occludin (OCLN), **b** claudin-1 (CLDN1), **c** zona occludens-1 (ZO1), and **d** haptogloblin/zonulin (zonulin). *p < 0.05

Tight junction gene expression was assessed in the ileum to determine if moderate intensity exercise induced intestinal barrier transcription alterations. Figure 2 demonstrates that OCLN and ZO1 gene expression was not changed (p > 0.05) but CLDN1 was decreased (p < 0.05) in the EXE group. Zonulin gene expression was higher in the EXE group compared to SED group (p < 0.05).

**Fig. 3:**
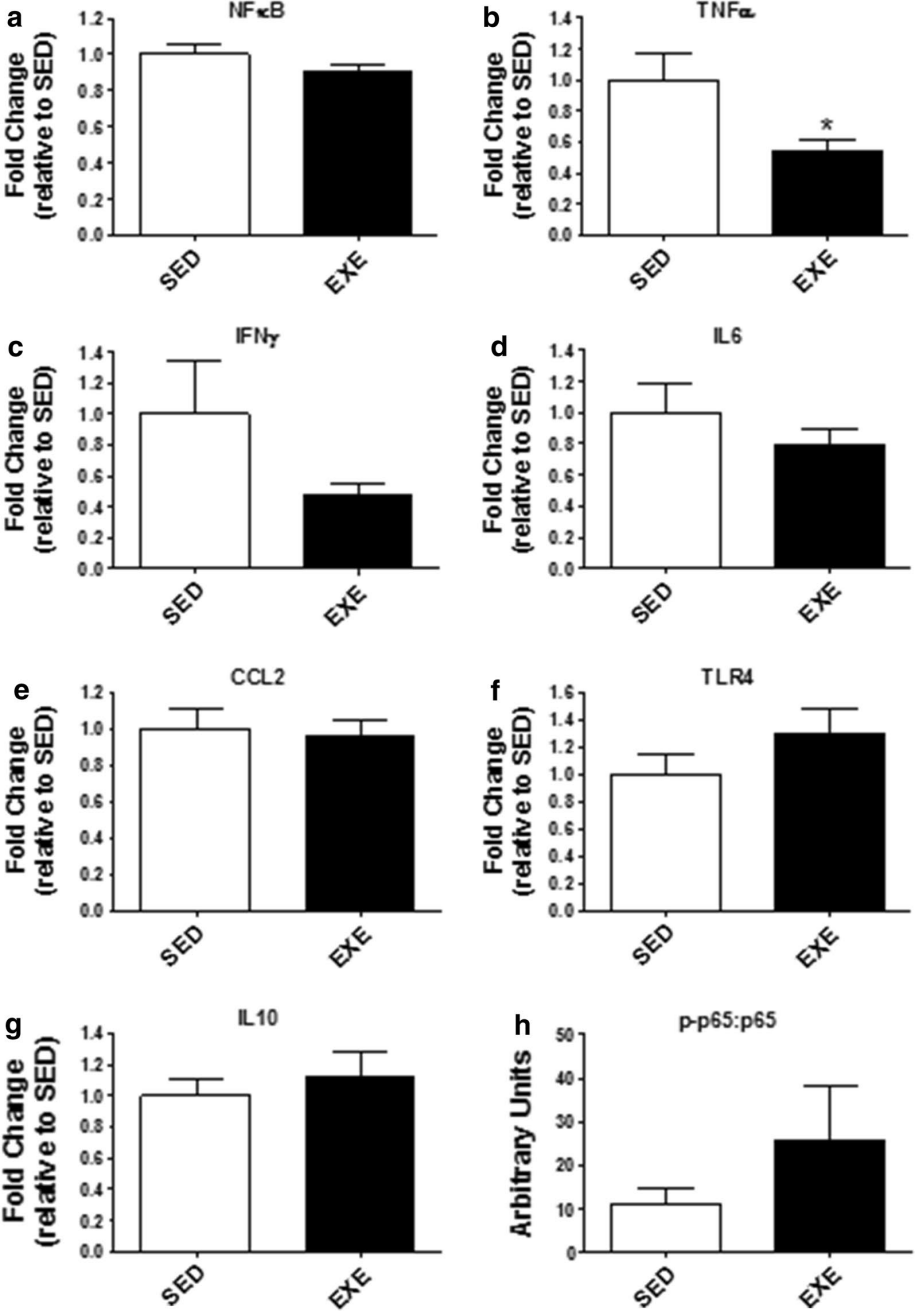
Ileum inflammatory markers. Gene expression of pro-inflammatory **a** nuclear factor kappa-B (NF_K_B), **b** tumor necrosis factor-α (TNFα), **c** interferon-γ (IFNγ), **d** interleukin 6 (IL6), **e** chemokine (C–C motif) ligand 2 (CCL2), and **f** toll-like receptor 4 (TLR4). Gene expression of anti-inflammatory **g** IL10 in the ileum. **h** protein levels of p-p65:p65. *p < 0.05

**Fig. 4:**
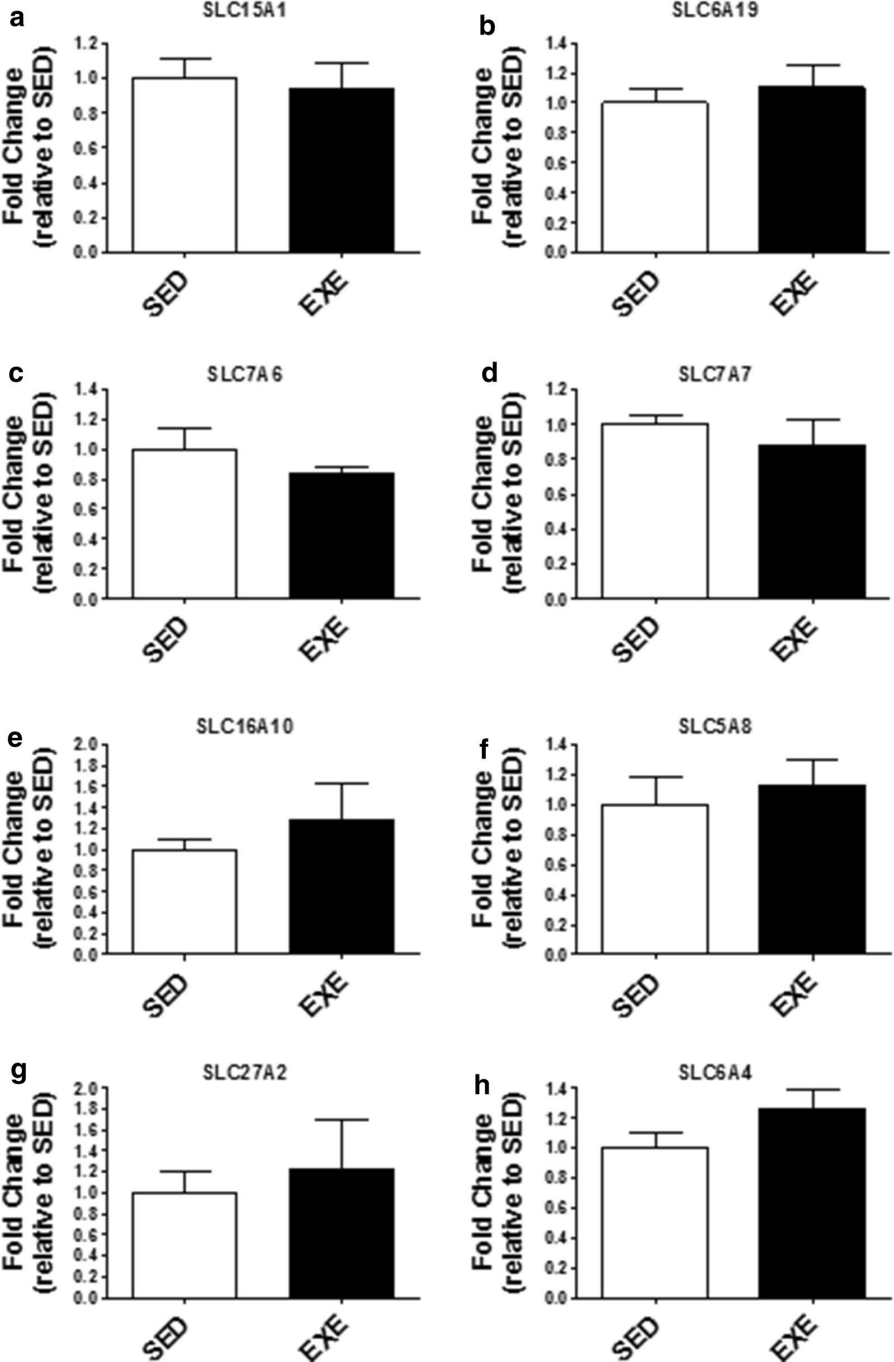
Gene expression of nutrient transporters in the ileum. **a** Oligopeptide transporter: PEPT1 (SLC15A1), **b** neutral amino acid transporter (SLC6A19), **c** amino acid transporter light chain, Y + L System (SLC7A6), **d** amino acid transporter light chain, Y + L System (SLC7A7), **e** aromatic amino acid transporter (SLC16A10), **f** lipid transporters: sodium/monocarboxylate cotransporter (SLC5A8), **g** fatty acid transporter (SLC27A2), and **h** neurotransmitter serotonin transporter (SLC6A4)

As illustrated in Fig. 3, the gene expression of TNFα was decreased (p < 0.05) in the EXE group; whereas the gene expression of other genes (NFκB, IFNγ, IL6, CCL2, and TLR4) were not altered by exercise (p > 0.05). Also, there was no change (p > 0.05) in p-p65:p65 protein levels. There was also no change (p > 0.05) in the gene expression of IL10 between SED and EXE groups. Similarly, mRNA expression patterns for select nutrient transporters, SLC15A1, SLC6A19, SLC7A6, SLC7A7, SLC16A10, SLC5A8, and SLC27A2, as well as the neurotransmitter serotonin transporter, SLC6A4, were not different (p > 0.05) between SED and EXE groups (Fig. 4).

## Discussion

Intestinal ischemia–reperfusion episodes during and post exercise are hypothesized as the culprit of exercise-induced gut distress [5] due to, among others factors, increased oxidant production [10, 11]. Increased hydrogen peroxide, generated from the dismutation of superoxide, can serve as signaling molecule that activates transcription of several genes including NF_K_B, TNFα, IL6, IFNγ, and IL1β [2]. As determined by the lipid peroxidation marker 4-HNE, our findings show no significant differences in oxidative damage between EXE and SED animals. An increase in antioxidant capacity without the concurrent change in oxidative stress provides evidence for an improved redox status. Our data demonstrate that ten consecutive days of moderate endurance exercise augments enzymatic antioxidant protein levels in the ileum 24 h after the final exercise bout, thereby improving ileum redox status. Hoffman-Goetz and collaborators found a similar response after 16 weeks of moderate endurance exercise, with significant increases of SOD1 and catalase in intestinal lymphocytes [12]. Furthermore, other studies have demonstrated a reduction in oxidative stress after both 10-55 days and 15 months of moderate endurance training compared to sedentary animals [13, 14]. Thus, endurance exercise at moderate intensities is a potential mechanism for providing the stimulus needed to increase ileum endogenous defense systems against oxidative tissue damage.

Intestinal barrier permeability may increase as a result of exercise-induced ischemia, but only when blood supply is reduced by at least 50 % [15]. More specifically, humans exercising at 70 % maximal oxygen consumption have a 60–70 % reduction in splanchnic blood flow [3] and at 100 % maximal oxygen consumption, an 80 % reduction [16]. We compared tight junction gene expression between EXE and SED animals and found that exercise increases zonulin mRNA and decreases CLDN1 mRNA with ZO1 and OCLN mRNA unaltered. CLDN1 and ZO1 are vital tight junction proteins and act to form the tight junction seal [17] while OCLN, a less vital tight junction protein, helps maintain the tight junction complex [18]. Zonulin, a protein responsible for the development of celiac disease, disrupts tight junctions to increase intestinal permeability in the jejunum and ileum [19, 20]. Our findings are inconsistent with previous literature as Teerapornpuntakit and collaborators demonstrated that 2 weeks of moderate endurance swimming for 60 min/day upregulated ZO1 and CLDN2 mRNA expression 1.5 fold in the ileum of rats (and 2-3 fold in the duodenum), thereby attesting the benefits of swimming on tight junction structure [21]. The differences observed between the two studies may be attributed to the fact that prolonged running may disrupt tight junction proteins via increased heat production in the intestinal wall coupled with ischemic/reperfusion stress [22, 23].

In regards to the effects of zonulin on tight junction protein expression, our findings are in partial agreement with the literature. Sapone and collaborators found increased intestinal permeability in type 1 diabetic patients [24]. Specifically, these authors report that serum zonulin levels were elevated with concomitantly increased CLDN1 mRNA, decreased CLDN2 mRNA and unaltered ZO1 and OCLN mRNA in the small intestine of type 1 diabetic subjects [24]. The portion of the small intestine examined in that study was not revealed; hence, that may explain our findings as tight junction mRNA expression differs with exercise in each section of the intestine [11, 21]. Collectively our data and the data of others suggest that an alteration in zonulin and CLDN1 with moderate intensity running may lead to increased intestinal permeability, thereby increasing the risk for endotoxin leakage from the lumen [8].

Finally, increased cytokine signaling may also act as a determinant of intestinal tight junction permeability [25]. An in vitro study demonstrated that increasing levels of TNFα, induced by NFκB, amplified intestinal epithelial tight junction permeability and down-regulated the expression of the tight junction protein ZO1 [25]. On the other hand, previous studies have shown that suppression of TNFα production and circulating levels occurs with regular exercise [26]. Therefore, our findings of reduced TNFα with unaltered NFκB and ZO1 mRNA expression are in agreement with current knowledge of transcriptional control by NFκB and TNFα. This reduction in intestinal inflammation 24 h post exercise may contribute to improved epithelial barrier function. To further elucidate the effects of 10 days of exercise training in the ileum, nutrient transporter mRNA levels were measured. Modification of nutrient transporter expression occurs in the intestines during periods of various stressors such as starvation, mental stress, and aging [27–29]. Our study shows that the gene expression of protein, fat, and carbohydrate transporters in the ileum does not change with moderate endurance exercise.

A limitation to our study includes the analysis of mRNA expression but not protein expression for the intestinal tight junction proteins as well as the marker of intestinal permeability. A second limitation includes the need to further assess permeability, possibly by measuring circulating lipopolysaccharide levels, to more accurately determine if the endurance exercise-induced increase in antioxidant enzymes helps maintain intestinal permeability. These analyses should be examined in future studies.

We conclude that 10 days of moderate intensity treadmill training in rats up-regulates key endogenous antioxidant enzymes, decreases select inflammation markers, and alters select markers of tight junction permeability in the ileum 24 h post exercise. Increased antioxidant production and decreased inflammation in the ileum resulting from sub-chronic endurance exercise may help to maintain normal intestinal permeability. The significance of our findings should be investigated further.

## Abbreviations

4-HNE: 4-hydroxynonenal
CCL2: chemokine (C–C motif) ligand 2
CLDN1: claudin 1
EXE: exercise
GI: gastrointestinal
GUSB: beta-glucuronidase
IFNγ: gamma interferon
IL10: interleukin 10
IL1β: interleukin 1β
IL6: interleukin 6
NFκB: nuclear factor kappa-B
OCLN: occludin
SED: sedentary
SLC15A1: solute carrier family 15 member 1 oligopeptide transporter
SLC16A10: solute carrier family 16 member 10 aromatic amino acid transporter
SLC27A2: solute carrier family 27 member 2 fatty acid transporter
SLC5A8: solute carrier family 5 member 8 sodium/monocarboxylate co-transporter
SLC6A19: solute carrier family 6 member 19 neutral amino acid transporter
SLC6A4: solute carrier family 6 member 4 sertonin neurotransmitter transporter
SLC7A6: solute carrier family 7 member 6 amino acid transporter light chain, y + L system
SLC7A7: solute carrier family 7 member 7 amino acid transporter light chain, y + L system
SOD2: superoxide dismutase 2
TLR4: toll-like receptor 4
TNFα: tumor necrosis factor-α
ZO1: zonula occluden-1
Zonulin: haptoglobin/zonulin
